# Supplementary material for: 3D integration enables ultralow-noise isolator-free lasers in silicon photonics
Source: Nature. 2023 Aug 2;620(7972):78–85. doi: 10.1038/s41586-023-06251-w (PMC10396957; doi:10.1038/s41586-023-06251-w)
Supplement: Supplementary file 1 — Supplementary Information [file 41586_2023_6251_MOESM1_ESM.pdf]

nature portfolio

https://doi.org/10.1038/s41586-023-06251-w

**Supplementary information**

# 3D integration enables ultralow-noise isolator-free lasers in silicon photonics

In the format provided by the authors and unedited

# Supplementary Information to:
# 3D integration enables ultra-low-noise isolator-free lasers in Si photonics

Chao Xiang[1,2,*,†], Warren Jin[1,3,*], Osama Terra[1,*,‡], Bozhang Dong[1,*], Heming Wang[1], Lue Wu[4], Joel Guo[1], Theodore J. Morin[1], Eamonn Hughes[5], Jonathan Peters[1], Qing-Xin Ji[4], Avi Feshali[3], Mario Paniccia[3], Kerry J. Vahala[4], and John E. Bowers[1,5,†]

[1]*Department of Electrical and Computer Engineering, University of California, Santa Barbara, CA, USA*
[2]*Department of Electrical and Electronic Engineering, The University of Hong Kong, Hong Kong, China*
[3]*Anello Photonics, Santa Clara, CA, USA*
[4]*T. J. Watson Laboratory of Applied Physics, California Institute of Technology, Pasadena, CA, USA*
[5]*Materials Department, University of California, Santa Barbara, CA, USA*
‡*Present address: Primary Length and laser technology lab, National Institute of Standards, Giza, Egypt*
*These authors contributed equally
†Email: cxiang@eee.hku.hk, bowers@ece.ucsb.edu

## I. 3D LAYER TRANSITION ILLUSTRATION

The full 3D mode transitions are illustrated in Supplementary Fig. 1. Four stages of vertical mode transitions are required to fulfill the InP/Si to SiN ULL mode transitions. In addition to the SiN RDL to SiN ULL taper loss we discussed above, the other three stages of taper losses are extensively studied in previous works [1–3]. The total taper loss should be below 1 dB in total thanks to the carefully designed adiabatic tapers and high-resolution mask alignment offered by the Deep-UV stepper ($<$ 100 nm misalignment accuracy).

## II. SURFACE QUALITY FOR HETEROGENEOUS BONDING

Fabrication of the ULL SiN layer and RDL SiN layer were performed at Tower Semiconductor, a commercial CMOS foundry (see Methods). As the final process step prior to the transfer of the samples from the foundry to the UCSB Nanofabrication facility, chemical-mechanical polishing (CMP) of the $SiO_2$ surface of the 200 mm wafer was performed. The CMP process resulted in a planarized surface with low surface roughness and excellent uniformity, which are key requirements for performing subsequent heterogeneous wafer bonding with high yield, and are a major benefit of working in a CMOS foundry. After coring the 200 mm wafer to a pair of 100 mm wafers, a silicon layer was bonded with a yield exceeding 95%, shown in Supplementary Fig. 2a. Such high yield is attributed to the excellent surface morphology of the $SiO_2$ surface resulting from the CMP process. Using an atomic-force microscope (Bruker ICON), the surface of the wafer prior to bonding was measured to exhibit a surface roughness of 0.2 nm r.m.s., shown in Supplementary Fig. 2b. The large-scale planarity of the wafer surface was also characterized using a surface stylus profilometer (Bruker DektakXT), as shown in Supplementary Fig. 2c. The full laser process is now partially performed in a CMOS fab for the FEOL SiN layers. With high-yield Si bonding, the BEOL Si and InP process is compatible with what Tower semiconductor is offering (PH18DA) [4], which shows the foundry compatibility of our devices that can support high-volume foundry productions in the future.

## III. LASER PERFORMANCE COMPARISON

The laser exhibits ultra-low frequency noise thanks to the ultra-high-Q SiN cavity. We compared the linewidth progression for heterogeneously integrated lasers in Supplementary Fig. 3. It can be seen that SiN cavities due to their ultra-low loss dominate heterogeneously integrated narrow linewidth laser performance and our device exhibits the current record linewidth performance. The demonstration of our platform which permits laser integration with ultra-low-loss SiN waveguides will soon lead to even better device performance. For example, using long waveguide spirals and micro-fabricated high-finesse mirrors with our 3D platform could demonstrate single-chip lasers with integrated linewidth on the order of 1 Hertz [5]. It has to be noted that the laser feedback insensitivity would allow end-fire coupling of the chip with micro-fabricated high-finesse cavities for such locking, which is another advantage of our platform in this direction.

Our approach offers the flexibility of microwave signal frequency tuning, which is hard to implement in approaches such as optical frequency comb-based microwave signal generation. The phase noise of the generated microwave signal

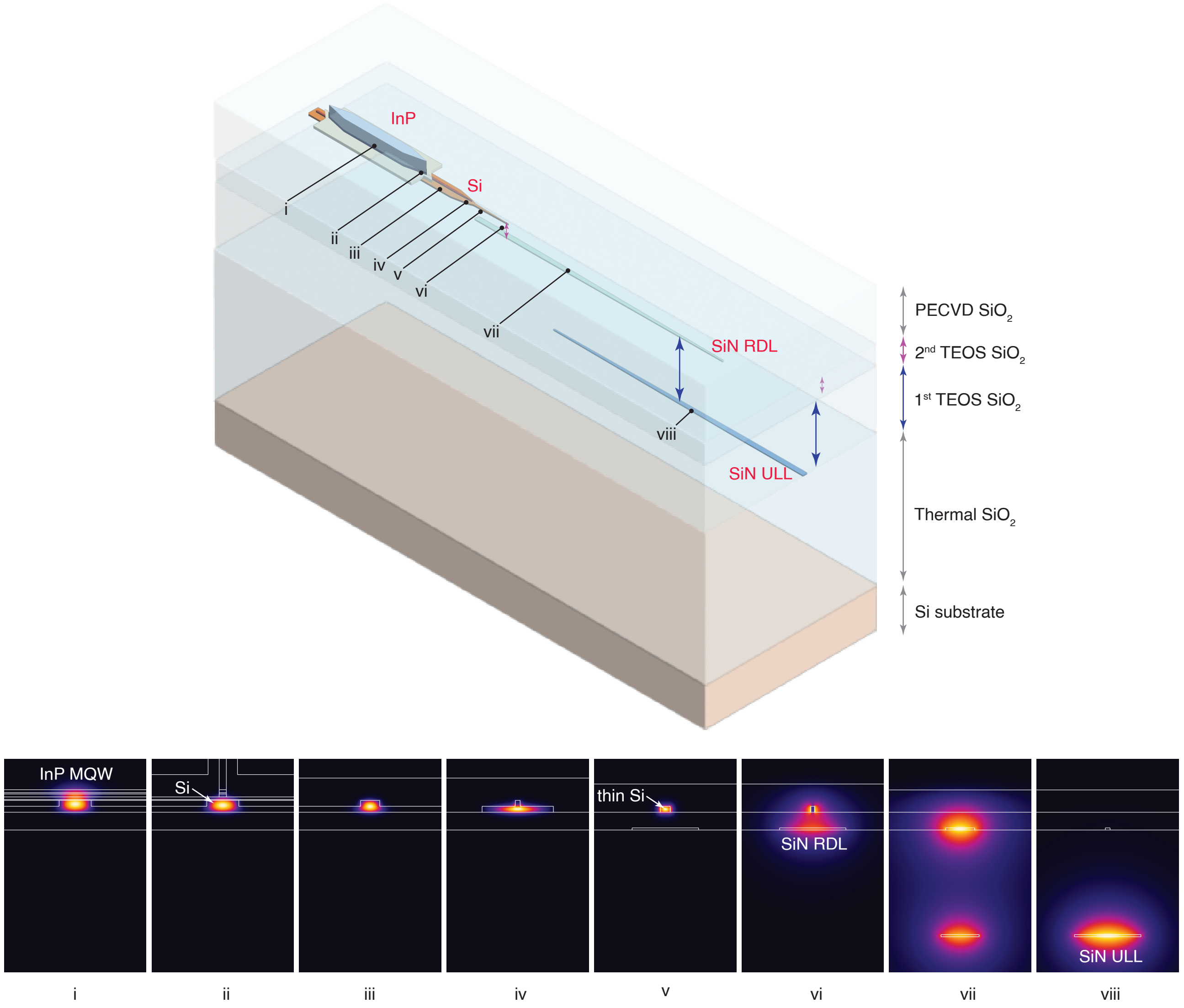


**Supplementary Figure** 1. **Mode transitions illustration and simulations**. The top figure shows the 3D structure of our devices with several tapers to fulfill the full 3D mode transitions including InP to Si (i to ii), Si to thin Si (iii to iv), thin Si to SiN RDL (v to vi), and SIN RDL to SiN ULL (vii to viii). The bottom panel shows the corresponding simulated mode profiles.

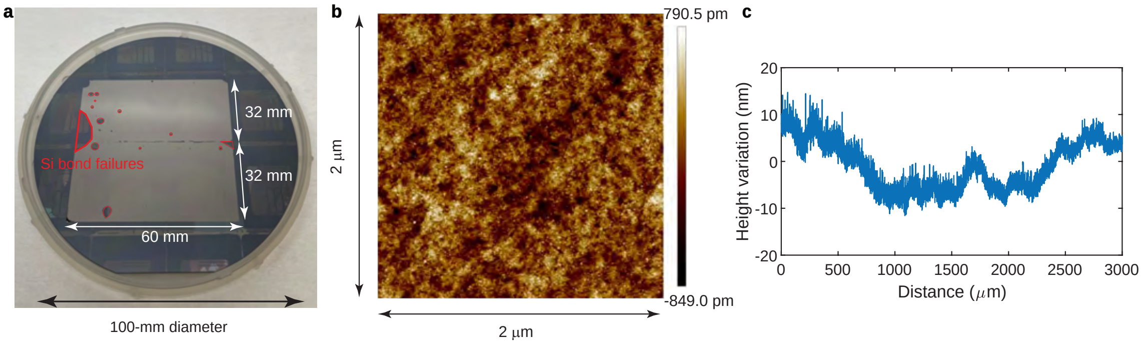


**Supplementary Figure** 2. **Si bonding yield and wafer smoothness data.** **a**. Wafer picture showing over 95% Si bonding yield. **b**. Wafer surface roughness. **c**. Wafer flat surface topographic variation.

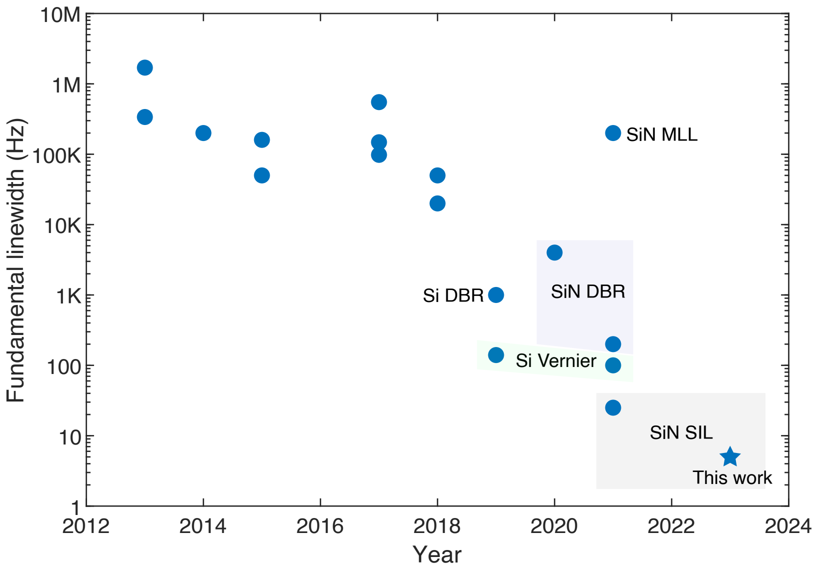


**Supplementary Figure 3. Comparison of the heterogeneously integrated laser linewidth performance.** The labeled references are Si DBR laser [6], Si Vernier lasers [7, 8], SiN MLL laser [9], SIN DBR lasers [3, 10] and SiN SIL laser [11].

is the sum of the phase noise of heterodyne beating lasers, which requires the beating lasers to be low-noise. As a comparison, optical frequency comb-based microwave generation benefits from the optical frequency division effect that could further reduce the signal phase noise once the noise is translated from the pump laser to the microwave signal [12]. So far, Kerr comb-based approach has achieved significant progress in generating low-noise microwave signals, but the direct on-chip laser integration for such a microwave-repetition-rate frequency comb generator has not been demonstrated yet. Mode-locked comb lasers can also serve as the source in microwave signal generation schemes, and a low-loss long passive cavity could improve the phase noise while reducing the comb spacing and microwave signal frequency. The tunability is also quite limited since it is mainly determined by the total cavity length. Table S1 compares the phase noise of our results with some of the recent demonstrations of photonic microwave generation.

| Platform | Approach | Frequency | Laser | Phase noise (dBc/Hz) | Laser linewidth |
|---|---|---|---|---|---|
| SiN [13] | Bright soliton | 20 GHz | Off-chip | -106 @ 10 kHz, -130 @ 100 kHz | NA |
| SiN [14] | Dark pulse | 10 GHz | Hybrid | -100 @ 10 kHz, -129 @ 100 kHz | 1.2 Hz |
| III-V/SiN [9] | Mode-locked laser | 755 MHz | On-chip | -85 @ 10 kHz, -108 @ 100 kHz | 200 kHz |
| III-V on Si [15] | Mode-locked laser | 20 GHz | On-chip | -67 @ 10 kHz, -85 @ 100 kHz | NA |
| III-V/Si [16] | Heterodyne | 0-50 GHz (Tunable, PD limited) | On-chip | NA | 150 kHz |
| This work | Heterodyne | 0-50 GHz (Tunable, PD limited) | On-chip | -53 @ 10 kHz, -83 @ 100 kHz | 5 Hz |

Table **S1**. Comparison of reported photonic microwave generation schemes.

## IV. DISCUSSION ON LASER LINEWIDTH IMPROVEMENT

The SiN resonator thermorefractive noise (TRN) currently dominantly limits the laser linewidth. To further reduce the laser linewidth, the approach is to extend the microresonator diameter to reduce the TRN, although this would increase the device footprint. Our device has a 30-GHz ring FSR that finds a balance in the laser linewidth and device footprint. However, our platform should be able to enable even lower laser linewidth thanks to the well-protected SiN waveguide low loss and resonator Q factors. To estimate the achievable laser fundamental linewidth using the current platform, we did the calculation based on the noise reduction factor (NRF) equation [14]:

$$\mathrm{NRF} \approx 4\left(1+\alpha^2\right) T^2 \eta^2 \frac{Q_\mathrm{R}^2}{Q_\mathrm{d}^2} \tag{1}$$

where $\alpha = 2.5$ is the laser linewidth enhancement factor, $T = 80\%$ is the laser to resonator coupling ratio, $\eta = 0.5$ is the bus waveguide to ring resonator coupling ratio, $Q_\mathrm{R} = 50 \times 10^6$ is the resonator loaded quality factor and $Q_\mathrm{d} = 10^4$ is the laser diode quality factor. The free-running fundamental laser linewidth is 800 kHz. The calculated laser fundamental linewidth is plotted in Supplementary Fig. 4. It can be seen that increasing the current 30-GHz-FSR resonator diameter to around 10-100 MHz could enable around 1 to 10 mHz laser fundamental linewidth using our platform. The low laser-to-resonator coupling loss benefits the linewidth reduction compared with hybrid integration schemes that normally suffer from a high coupling loss.

Increasing the resonator radius (or circumference) to reduce the TRN will lead to lower FSR. It needs to be noted that, in ring-resonator-based Vernier lasers, extended ring circumference can lead to narrow longitudinal mode spacing that gives rise to severe mode hops. For the self-injection locking scheme in our work, the mode-hop-free tuning of the lasing wavelength can be achieved by the simultaneous tuning of the DFB lasing wavelength by the gain current tuning and the ring resonance by heater tuning at the same rate. Such requirement is independent of the resonator diameter or FSR. As a result, the increase of the ring circumference will not increase the chance of mode-hops as in the case of Vernier ring lasers. Integrating ultra-high-Q resonators for ultra-narrow-linewidth lasers with Hertz-linewidth or lower is paramount for applications such as ultra-low-noise microwave synthesis, precision sensors, atomic clocks, and so on. These applications put a more stringent requirement on the laser linewidth than conventional integrated semiconductor lasers could offer.

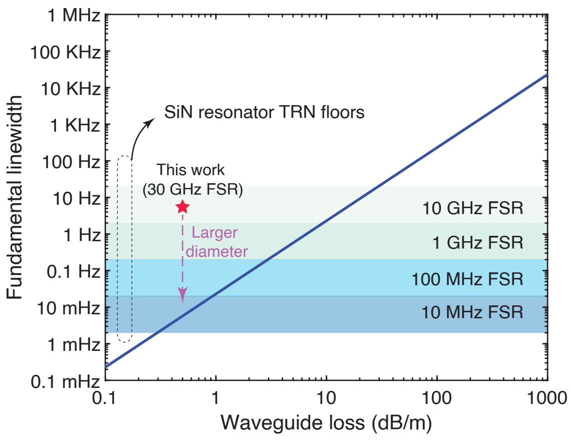


**Supplementary Figure** 4. **Achievable laser linewidth estimation.** The laser fundamental linewidth estimation from its noise reduction factor due to the laser self-injection locking. The shaded color areas show the estimated TRN floors for different resonator FSRs. For each FSR, the TRN floor used for fundamental linewidth estimation can vary by about ten times depending on the offset frequency that the frequency noise trace starts to flatten out to its white noise level.

## V. SELF-INJECTION LOCKING RANGE

The injection-locked laser frequency, where a high-$Q$ cavity is providing the reflection, can be described by the following implicit equation [17]:

$$\delta_\mathrm{L} = \delta + K \frac{2\delta\cos\phi + (1+\beta^2-\delta^2)\sin\phi}{(1+\beta^2-\delta^2)^2 + 4\delta^2} \tag{2}$$

where $\delta_\mathrm{L} = 2\delta\omega_\mathrm{L}/\kappa$ is the normalized detuning for the free-running laser with respect to the high-$Q$ cavity, $\delta = 2\delta\omega/\kappa$ is the normalized detuning for the injection-locked laser, $\kappa$ is the resonator loss rate, $\beta$ is the on-resonance amplitude reflection ratio, $\phi$ is the roundtrip feedback phase, $K = 4\sqrt{1+\alpha_\mathrm{H}^2}\eta\beta\kappa_\mathrm{L}/\kappa$ is the normalized locking bandwidth, $\alpha_\mathrm{H}$ is the laser linewidth enhancement factor, $\eta$ is the coupling ratio for the cavity, and $\kappa_\mathrm{L}$ is the laser out-coupling rate. If

the backscattering is weak ($|\beta| \ll 1$), then the mode splitting effect in the feedback lineshape can be ignored, leading to a simplified expression:

$$\delta_{\rm L} \approx \delta + K\frac{2\delta\cos\phi + (1-\delta^2)\sin\phi}{(1+\delta^2)^2} \tag{3}$$

When $\phi = 0$, $\delta$ is an odd function of $\delta_{\rm L}$, and the locking curve is symmetric. In this case, the maximum range that the laser remains locked can be found as, assuming that $K \gg 1$,

$$\delta_{\rm L,Locked} \approx 2K \max_{\delta} \frac{2\delta}{(1+\delta^2)^2} = \frac{3\sqrt{3}}{4}K \tag{4}$$

or in physical units, $\delta\omega_{\rm L,Locked} = 3\sqrt{3}\sqrt{1+\alpha_{\rm H}^2}\eta\beta\kappa_{\rm L}/2$. However, when the laser (or the resonator) is sweeping its frequency, the locking range could be smaller than this number. As the laser frequency approaches the resonance, it remains in a metastable unlocked state until the locked state is the only state allowed for the current laser-resonator detuning. For realistic lasers, the existence of relaxation frequency in the gain sections may excite sidebands at the corresponding frequency. The laser may be pulled to the locked state in advance when the laser-resonator detuning is close to the relaxation frequency, and the locking range is increased accordingly. If the feedback phase is different from 0, the feedback lineshape becomes asymmetric, which also affects the locking range when sweeping in both directions. Such asymmetric locking range is clear as shown in Supplementary Fig. 5.

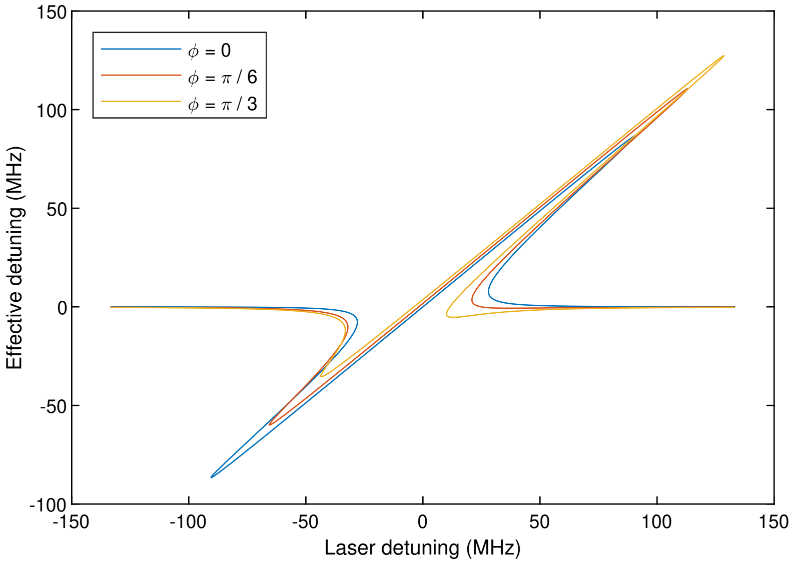


**Supplementary Figure** 5. **Mode frequencies of the injection-locked laser as predicted by Eq.(4).** Here the values of the feedback phase $\phi$ are 0, $\pi/6$ and $\pi/3$. Increased $\phi$ leads to asymmetry of the locking range. The normalized locking bandwidth is taken as $K = 20$. This value is significantly smaller than the experimentally obtained value ($K \approx 243$ as in Fig. 2d in the main text) and is chosen to demonstrate the overall locking curve shape.

## VI. METHODS TO MEASURE THE LASER FREQUENCY NOISE

There are various methods to measure the frequency noise of a laser. Two of the methods used here are demonstrated in Supplementary Fig. 6. In the homodyne method, the laser input goes through a Mach-Zehnder interferometer and the output is detected. Depending on the noise level of the laser and the MZI FSR, light in the two arms may be mostly coherent or almost incoherent. For low-noise lasers, it is impractical for the arm length difference to exceed the laser coherent length, and the homodyne method always operates in the coherent regime, where the MZI output follows its transfer function in its steady state. In this case, the frequency variation of the laser shows up as voltage changes in the electric output, and the laser frequency noise spectrum can be calculated from the DC-band noise spectrum of the voltage signal. To maximize the response of the system, the phase of the MZI is tuned to be at the quadrature point, and additional locking is required if the long-term stability of the laser causes the system to drift away from the quadrature point.

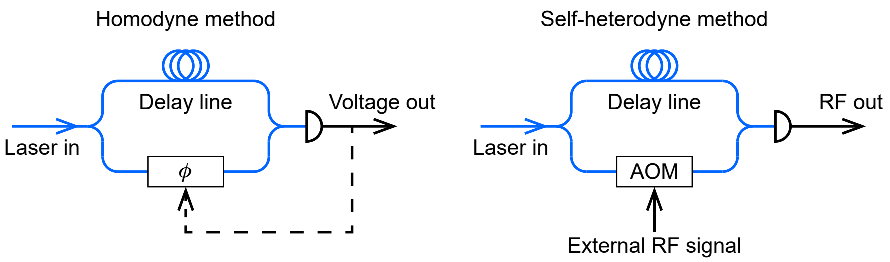


**Supplementary Figure** 6. **Schematics of two possible frequency noise measurement methods.** Left: the homodyne method consists of an MZI structure, where the phase of one arm is adjustable for quadrature point tuning. An external locking loop can be added to control this phase. Right: the self-heterodyne method is similar but includes a frequency shifter in one arm. No quadrature point tuning or locking is required. AOM, acousto-optic modulator.

The self-heterodyne method works similarly to the homodyne method, except that one arm now includes a frequency shifter (typically an acousto-optic modulator) so that the light from two arms now has different frequencies. The beating of these signals produces an RF-domain signal, and any frequency variation of the laser will show up as changes in the RF frequency. The laser frequency noise spectrum can then be recovered from the phase noise of the RF signal. The use of RF signals eliminates the locking procedure as well as the low-frequency noise contributions from the electronics, and the measurement is no longer limited by the MZI FSR. Balanced detectors for both MZI outputs can be used to minimize the influence of intensity noise, and two identical setups can run in parallel for cross-correlation to further reduce the detection noise [18].

## VII. FEEDBACK SENSITIVITY FOR SEMICONDUCTOR LASERS

In general, semiconductor laser diodes exhibit strong instability under external optical feedback due to their class B nature. The classification of a laser system depends on the photon lifetime $\tau_p$, the carrier lifetime $\tau_c$ and the polarization lifetime $\tau_{pol}$. For a class B laser, it should obey $\tau_c \geq \tau_p \gg \tau_{pol}$. As explained in Ref. [19], an additional degree of freedom such as Q-switching, optical injection, and optical feedback is able to trigger some unstable dynamics from those lasers. The process of back reflection is to send part of the laser's emitted field back to the laser cavity. Therefore, two important parameters are related to this process. The first one is the feedback strength, whose definition has been introduced in Eq. (1) in the main text. The other one is the external cavity length $L_{ext}$ that corresponds to the external round-trip time $\tau_{ext}$, which is expressed as:

$$\tau_{ext} = \frac{2L_{ext}}{v_g} \tag{5}$$

where $v_g$ denotes the group velocity in the external cavity.

When a semiconductor laser is subject to external optical feedback, the amplitude-phase coupling between the returned field and the intra-cavity one is represented by the field fluctuations in amplitude and phase. The perturbation of the photon density due to the re-injected field will lead to a fluctuation of both the carrier density and the optical gain. The latter, which is attributed to the damping effect, changes the refractive index through the linewidth enhancement factor ($\alpha_H$-factor), which results in a change in the lasing wavelength. On the other hand, the returned field will directly lead to a wavelength fluctuation through the phase fluctuation. As a result, the interaction of the intensity and the phase results in complex nonlinear dynamics when the laser is subject to back reflection. Severe optical instabilities such as periodic oscillation, coherence collapse, or low-frequency fluctuations can be triggered [20].

To analyze the feedback sensitivity of laser diode, N. Schunk and K. Petermann proposed the C-parameter[21], which is expressed as follows:

$$C = \frac{\tau_{ext}}{\tau_{in}} 2C_l \sqrt{1+\alpha_H^2}\sqrt{\eta_F} \tag{6}$$

in which $\tau_{in}$ and $C_l$ are the round-trip time of the laser cavity resonance and the coupling coefficient from the laser to the external cavity, respectively. The latter can be roughly determined by $C_l$=$(1-R)/\sqrt{R}$ where R accounts for the facet power reflection.

Depending on the feedback strength and the external cavity length, Tkach and Chraplyvy identified five feedback regimes for a DFB laser diode [22]. A laser diode is unconditionally stable against back reflection in Regime I where $C \leq 1$. With the increase of feedback strength or the external cavity length that makes $C > 1$, due to the phase fluctuation and mode hopping caused by the returned field, the optical linewidth of the laser is either narrowed or broadened in Regime II. In this work, the feedback sensitivity of the laser is analyzed in a strict condition by using an 8-meter-long optical fiber as the external cavity loop, which provides significantly more reflection than that of on-chip reflections. It is worth stressing that the laser will be much more robust against back reflections when the external cavity length is reduced to a few centimeters, which is the case that a laser encounters on chip [23]. A laser can be restabilized by the optical feedback in Regime III, where the optical linewidth is reduced. Once a laser operates above Regime II, its dynamics will no longer depend on the external cavity length. In Regime IV where the feedback strength is strong enough, coherence collapse happens. In this regime, the laser at first potentially exhibits two frequencies with an incommensurable ratio at the relaxation oscillation frequency and at the external cavity frequencies. As shown in the left figure in Fig. 3d in the main text, the coherence of the free-running laser is not fully collapsed as the feedback strength increases from -15 to -13 dB. Nevertheless, the laser will definitely experience coherence collapse under a stronger feedback strength above -13 dB. Some unstable laser sources, i.e., bulk laser diodes, can enter into Regime V if the feedback strength can be further improved. In this regime, the laser will be highly stable and shows a single narrow-linewidth longitudinal mode that is determined by the external cavity frequency. Nevertheless, this regime is very difficult to reach since the feedback strength is usually limited by the facet reflection.

To develop an on-chip source that is tolerant to back reflections, one of the most efficient ways is to decouple the interaction between the intensity and the phase in the active region. In other words, using a gain medium with near-zero $\alpha_H$-factor like semiconductor quantum-dots (QD) [24], as we can see from Eq. (6). Another way is to decouple the reflected field and the intra-cavity field by increasing the quality factor of the laser cavity, which is the objective of this work. As one can see from Eq. (6), the C-parameter is determined by the coupling ratio of the laser cavity and the external reflected field. Such a coupling ratio is inversely proportional to the Q-factor of the laser cavity. To have a quantitative analysis of the feedback sensitivity of a laser with different Q-factor, the C-parameter can be rewritten as follows [25]:

$$C = \frac{\omega_0 \tau_{ext}}{Q} \sqrt{1 + \alpha_H^2} \sqrt{\eta_F} \tag{7}$$

with $\omega_0$ the angular frequency of the laser emission. It should be noted that Eq. (7) is valid under the assumption that the high-Q laser has a perfect power reflection approaching unity hence the Q-factor can be directly described as a function of the transmission loss. On the other hand, according to Eq. (7), a long external cavity such as 8 meters in this study would largely increase the C-parameter and easily make it above unity. Nevertheless, it holds when the feedback operation is in the short-cavity regime, where the external cavity frequency is higher than the relaxation oscillation frequency $f_r$ of the laser [23]. Once the external round-trip time exceeds the relaxation time of the laser, the feedback sensitivity becomes less dependent on the external cavity length and the C-parameter.

To analyze the feedback sensitivity of QW laser, a single-frequency stability equation that is derived from K. Petermann's work is expressed as follows [25, 26]:

$$f(\Delta\omega\tau_{ext}) = \Delta\omega\tau_{ext} + C sin(\Delta\omega\tau_{ext} + \omega_0\tau_{ext} + tan^{-1}\alpha_H) \tag{8}$$

with

$$\Delta\omega\tau_{ext} = (\omega - \omega_0)\tau_{ext} \tag{9}$$

Here, Eq. (8) accounts for the laser phase variation due to the external back reflection. When the QW laser is stable against the reflection in the presence of a coherent single-frequency emission, there is only one solution for $f(\Delta\omega\tau_{ext})$=0. Nevertheless, the coherence collapse takes place in a severe feedback condition where the C-parameter is above unity. As a result, multimode instability is triggered by the back reflection, and an increase of the number of solutions for $f(\Delta\omega\tau_{ext})$=0 will be observed. Supplementary Figure 7 depicts the calculated $f(\Delta\omega\tau_{ext})$ as a function of $\Delta\omega\tau_{ext}$ for a QW laser when the feedback strength is -20 dB. For the QW laser in this study, $\omega_0/2\pi = 193$ THz, $f_r = 2$ GHz, and $\alpha_H = 2.5$. Eq. (7) is still valid for analyzing the feedback sensitivity by assigning 50 cm to the fiber external cavity loop. For QW laser with a Q-factor at $5\times10^4$ (black) or $1\times10^5$ (blue), the corresponding C-parameter is 16.4 and 8.2, respectively. The multimode instability due to back reflection can be seen from the non-zero solutions for $f(\Delta\omega\tau_{ext})$=0, which is the reason why QW laser is very feedback-sensitive and cannot tolerate an external reflection as strong as -20 dB. Nevertheless, the laser can be much more tolerant to the back reflections as the Q-factor increases to $1\times10^6$ (yellow) or even to $10\times10^6$ (red), where the C-parameter is reduced to 0.82 and 0.082, respectively. Therefore, the coherence of the QW laser can be maintained under a strong chip-scale back reflection by taking advantage of the great Q-factor from the microresonator.

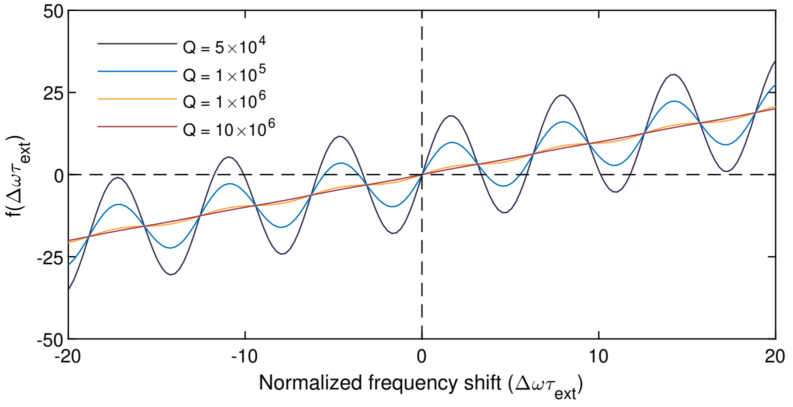


**Supplementary Figure 7. Dependence of steady-state solutions for the phase on the Q-factor.** Under a feedback strength at -20 dB, the Q-factor of the QW laser increases from $5\times10^4$ (black), $1\times10^5$ (blue), $1\times10^6$ (yellow), to $10\times10^6$ (red). The external round-trip length is 50 cm in fiber.

As the feedback sensitivity is determined by a C-parameter [21] and the laser coherence will be maintained as long as the C-parameter is below unity. It should be noted that the C-parameter is proportional to the external cavity delay time, which means that the laser will suffer from a much more severe feedback condition when the external cavity is long. In our study, the 8-meter-long optical fiber used as the external cavity provides more stringent constraints on analyzing the feedback sensitivity than short-distance on-chip reflections. The transition from Regime I to Regime II will be largely delayed by reducing the external cavity length into the short-cavity regime, such as the case for on-chip integration [23].

In Table S2, we list the performance comparison of the current reflection insensitive sources. It should be noted here that there are no standards for isolator-free operation on PICs, so we cannot make a fair comparison between our laser and other solutions. The only standard for fiber-optic systems is IEEE 802.3 standard. A laser source must tolerate at least -21 dB off-chip feedback strength in the fiber-optic system. Nevertheless, this standard is not highly effective for PICs, since the unaccounted fiber-chip coupling loss and the chip-chip coupling loss determine how much power is resent into the laser cavity. In this context, a criterion that includes the coupling loss remains to be developed. On the other hand, the requirement of feedback insensitivity is dependent on the applications. As aforementioned, the laser maintains its coherence in feedback regime II, in which the laser linewidth is either narrowed or broadened. Despite the fact that one can benefit from a lower frequency noise in regime II, the uncontrolled linewidth fluctuation would be detrimental to a system that requires an ultra-stable operation. Therefore, a strict criterion for isolator-free

| Wavelength | Platform | Design | Critical feedback level | | Frequency noise comparison | Ref. No. |
|---|---|---|---|---|---|---|
| | | | Off-chip | On-chip | | |
| 1.55 $\mu m$ | QW | DFB + high Q resonator | $>$ -0.9 dB | $>$ -6.9 dB | Yes | This work |
| 1.55 $\mu m$ | QW | DFB | -31 dB | N/A | Yes | [27] |
| 1.49 $\mu m$ | QW | DFB | -9 dB | N/A | No | [28] |
| 1.55 $\mu m$ | QW | DFB | -6 dB | N/A | No | [29] |
| 1.56 $\mu m$ | QW | DFB | N/A | -14 dB$^\dagger$ | No | [30] |
| 1.59 $\mu m$ | QDash | FP | N/A | -23 dB$^\dagger$ | No | [31] |
| 1.31 $\mu m$ | QW | DBR | -4 dB | N/A | No | [32] |
| 1.3 $\mu m$ | QD | FP | N/A | $>$ -7.4 dB$^\dagger$ | No | [24] |
| 1.3 $\mu m$ | QD | FP | N/A | -3.5 dB$^*$ | No | [23] |
| 1.3 $\mu m$ | QD | DFB | N/A | -6 dB$^\dagger$ | No | [33] |

$^\dagger$ Only the forward fiber coupling loss is included. Some of these studies defined the boundary of regime IV as the critical feedback level, which excluded the influence of linewidth fluctuation in regime II.

$^*$ Back reflection in the short-cavity regime, where the relaxation oscillation frequency is lower than the external cavity frequency.

Table **S2**. Performance comparison of reflection-insensitive semiconductor lasers.

operation is crucial for ultra-low-noise microwave synthesis, precision sensors, atomic clocks, etc. In this work, the calculation of feedback strength includes both the forward and the backward fiber-chip coupling loss, and the critical feedback level is defined as the boundary of regime I in which the laser dynamics are unaffected. As shown in Fig. 3 in the main text, the ultra-low laser linewidth is maintained when the on-chip feedback strength is as large as -6.9 dB, where the injection-locked DFB laser still operates in regime I. For some relative studies on QW lasers[28, 29, 32], the feedback strength only considers the loss of the off-chip feedback loop, which does not correspond to the case of PIC. In recent years, QD lasers are regarded as a promising solution for isolator-free applications, owing to their near-zero $\alpha_H$-factor. Nevertheless, one cannot determine if the lasers are operating in regime II due to the absence of the frequency noise comparison in some relative studies[23, 24, 33]. Therefore, a comparison of the laser frequency noise between with and without optical feedback is critical to determine the stability of laser operation. Even if we apply the strictest criterion, the reflection insensitivity of our laser is still on par with or even better than the most advanced QD laser. Another relative work on QW laser characterized its reflection sensitivity by comparing the frequency noise spectrum[27] and the laser starts to exhibit instability with off-chip feedback strength of -31 dB. In comparison, our device still maintains similar laser frequency noise with off-chip reflection strength as high as -0.9 dB.

## VIII. FEEDBACK SENSITIVITY DEPENDENCE ON Q FACTOR

In the presence of an external high-$Q$ cavity that provides narrow-linewidth reflection, the robustness of the composite laser with respect to other feedback sources is increased. This can be understood as an increase in the local stability of the injection-locked mode in question. Specifically, the large phase response provided by the cavity could tolerate more phase disturbances from additional reflections to the laser. Since the $C$ parameter can also be interpreted as the slope of the frequency solution curve induced by the feedback (e.g. Supplementary Fig. 7), this effect can be described with a model analogous to the previous section by including the contribution of intentional feedback in the $C$ parameter. This mechanism determines the increase of feedback tolerance in the low-$Q$ cavity regime (as shown in Fig. 3c in the main text).

At larger cavity $Q$ factors, the relative amplitude of cavity reflection and external reflection also becomes important. If the external reflection has a larger amplitude, it dominates over the reflected signal from inside the cavity, and the unstable phase from the external reflection makes the laser lose stability. This shows up in the frequency solution curve as the bifurcation of modes when the reflection phase is changing. The injection-locked mode may disappear at certain reflection phases if the external reflection is large enough. In this case, the tolerable reflection is almost independent of the $Q$ factor, as it is instead determined by the amplitude of cavity reflection. This observation agrees with numerical calculations, and the plateau at high-$Q$ factors shown in Fig.3c in the main text is determined by this mechanism.

---


[1] M. L. Davenport, S. Skendžić, N. Volet, J. C. Hulme, M. J. Heck, and J. E. Bowers, Heterogeneous silicon/III–V semiconductor optical amplifiers, IEEE Journal of Selected Topics in Quantum Electronics **22**, 78 (2016).

[2] J. F. Bauters, M. L. Davenport, M. J. Heck, J. Doylend, A. Chen, A. W. Fang, and J. E. Bowers, Silicon on ultra-low-loss waveguide photonic integration platform, Optics Express **21**, 544 (2013).

[3] C. Xiang, W. Jin, J. Guo, J. D. Peters, M. J. Kennedy, J. Selvidge, P. A. Morton, and J. E. Bowers, Narrow-linewidth III-V/Si/$Si_3N_4$ laser using multilayer heterogeneous integration, Optica **7**, 20 (2020).

[4] OpenLight demos 224G InP-based modulator for Tower's PH18DA platform, `https://www.semiconductor-today.com/news_items/2023/mar/openlight-070323.shtml`, 7 March 2023.

[5] J. Guo, C. A. McLemore, C. Xiang, D. Lee, L. Wu, W. Jin, M. Kelleher, N. Jin, D. Mason, L. Chang, A. Feshali, M. Paniccia, P. T. Rakich, K. J. Vahala, S. A. Diddams, F. Quinlan, and J. E. Bowers, Chip-based laser with 1-hertz integrated linewidth, Science Advances **8**, eabp9006 (2022).

[6] D. Huang, M. A. Tran, J. Guo, J. Peters, T. Komljenovic, A. Malik, P. A. Morton, and J. E. Bowers, High-power sub-kHz linewidth lasers fully integrated on silicon, Optica **6**, 745 (2019).

[7] M. A. Tran, D. Huang, and J. E. Bowers, Tutorial on narrow linewidth tunable semiconductor lasers using Si/III-V heterogeneous integration, APL photonics **4**, 111101 (2019).

[8] P. A. Morton, chaoxiang, J. Khurgin, C. D. Morton, M. A. Tran, J. D. Peters, J. Guo, M. J. Morton, and J. E. Bowers, Integrated Coherent Tunable Laser (ICTL) with ultra-wideband wavelength tuning and sub-100 Hz Lorentzian linewidth, Journal of Lightwave Technology (2021).

[9] S. Cuyvers, B. Haq, C. Op de Beeck, S. Poelman, A. Hermans, Z. Wang, A. Gocalinska, E. Pelucchi, B. Corbett, G. Roelkens, *et al.*, Low Noise Heterogeneous III-V-on-Silicon-Nitride Mode-Locked Comb Laser, Laser & Photonics Reviews , 2000485 (2021).

[10] C. Xiang, J. Guo, W. Jin, L. Wu, J. Peters, W. Xie, L. Chang, B. Shen, H. Wang, Q.-F. Yang, D. Kinghorn, M. Paniccia,

K. J. Vahala, P. A. Morton, and J. E. Bowers, High-performance lasers for fully integrated silicon nitride photonics, Nature Communications **12**, 6650 (2021).

[11] C. Xiang, J. Liu, J. Guo, L. Chang, R. N. Wang, W. Weng, J. Peters, W. Xie, Z. Zhang, J. Riemensberger, J. Selvidge, T. J. Kippenberg, and J. E. Bowers, Laser soliton microcombs heterogeneously integrated on silicon, Science **373**, 99 (2021).

[12] T. M. Fortier, M. S. Kirchner, F. Quinlan, J. Taylor, J. C. Bergquist, T. Rosenband, N. Lemke, A. Ludlow, Y. Jiang, C. W. Oates, and S. A. Diddams, Generation of ultrastable microwaves via optical frequency division, Nature Photonics **5**, 425 (2011).

[13] J. Liu, E. Lucas, A. S. Raja, J. He, J. Riemensberger, R. N. Wang, M. Karpov, H. Guo, R. Bouchand, and T. J. Kippenberg, Photonic microwave generation in the X-and K-band using integrated soliton microcombs, Nature Photonics **14**, 486 (2020).

[14] W. Jin, Q.-F. Yang, L. Chang, B. Shen, H. Wang, M. A. Leal, L. Wu, M. Gao, A. Feshali, M. Paniccia, K. J. Vahala, and J. E. Bowers, Hertz-linewidth semiconductor lasers using CMOS-ready ultra-high-Q microresonators, Nature Photonics **15**, 346 (2021).

[15] D. Auth, S. Liu, J. Norman, J. E. Bowers, and S. Breuer, Passively mode-locked semiconductor quantum dot on silicon laser with 400 Hz RF line width, Optics Express **27**, 27256 (2019).

[16] J. Hulme, M. Kennedy, R.-L. Chao, L. Liang, T. Komljenovic, J.-W. Shi, B. Szafraniec, D. Baney, and J. E. Bowers, Fully integrated microwave frequency synthesizer on heterogeneous silicon-III/V, Opt. Express **25**, 2422 (2017).

[17] N. M. Kondratiev, V. E. Lobanov, A. V. Cherenkov, A. S. Voloshin, N. G. Pavlov, S. Koptyaev, and M. L. Gorodetsky, Self-injection locking of a laser diode to a high-Q WGM microresonator, Optics Express **25**, 28167 (2017).

[18] Z. Yuan, H. Wang, P. Liu, B. Li, B. Shen, M. Gao, L. Chang, W. Jin, A. Feshali, M. Paniccia, *et al.*, Correlated self-heterodyne method for ultra-low-noise laser linewidth measurements, Optics Express **30**, 25147 (2022).

[19] F. Arecchi, R. Meucci, G. Puccioni, and J. Tredicce, Experimental evidence of subharmonic bifurcations, multistability, and turbulence in a Q-switched gas laser, Physical Review Letters **49**, 1217 (1982).

[20] J. Ohtsubo, *Semiconductor lasers: stability, instability and chaos*, Vol. 111 (Springer, 2012).

[21] N. Schunk and K. Petermann, Numerical analysis of the feedback regimes for a single-mode semiconductor laser with external feedback, IEEE Journal of Quantum Electronics **24**, 1242 (1988).

[22] R. Tkach and A. Chraplyvy, Regimes of feedback effects in 1.5-µm distributed feedback lasers, Journal of Lightwave Technology **4**, 1655 (1986).

[23] B. Dong, J.-D. Chen, F.-Y. Lin, J. C. Norman, J. E. Bowers, and F. Grillot, Dynamic and nonlinear properties of epitaxial quantum-dot lasers on silicon operating under long-and short-cavity feedback conditions for photonic integrated circuits, Physical Review A **103**, 033509 (2021).

[24] J. Duan, H. Huang, B. Dong, D. Jung, J. C. Norman, J. E. Bowers, and F. Grillot, 1.3-$\mu m$ Reflection Insensitive InAs/GaAs Quantum Dot Lasers Directly Grown on Silicon, IEEE Photonics Technology Letters **31**, 345 (2019).

[25] F. Grillot, J. C. Norman, J. Duan, Z. Zhang, B. Dong, H. Huang, W. W. Chow, and J. E. Bowers, Physics and applications of quantum dot lasers for silicon photonics, Nanophotonics **9**, 1271 (2020).

[26] K. Petermann, External optical feedback phenomena in semiconductor lasers, IEEE Journal of Selected Topics in Quantum Electronics **1**, 480 (1995).

[27] Z. Zhang, K. Zou, H. Wang, P. Liao, N. Satyan, G. Rakuljic, A. E. Willner, and A. Yariv, High-speed coherent optical communication with isolator-free heterogeneous Si/III-V lasers, Journal of Lightwave Technology **38**, 6584 (2020).

[28] M. Moehrle, W. Brinker, C. Wagner, G. Przyrembel, A. Sigmund, and W. Molzow, First complex coupled 1490nm CSDFB lasers: High yield, low feedback sensitivity, and uncooled 10Gb/s modulation, in *2009 35th European Conference on Optical Communication* (IEEE, 2009) pp. 1–2.

[29] V. Brac de la Perrière, Q. Gaimard, H. Benisty, A. Ramdane, and A. Lupu, Electrically injected parity-time symmetric distributed feedback laser diodes (DFB) for telecom applications, Nanophotonics **10**, 1309 (2021).

[30] S. Gomez, H. Huang, J. Duan, S. Combrié, A. Shen, G. Baili, A. de Rossi, and F. Grillot, High coherence collapse of a hybrid III–V/Si semiconductor laser with a large quality factor, Journal of Physics: Photonics **2**, 025005 (2020).

[31] B. Dong, J. Duan, C. Shang, H. Huang, A. B. Sawadogo, D. Jung, Y. Wan, J. Bowers, and F. Grillot, Influence of the polarization anisotropy on the linewidth enhancement factor and reflection sensitivity of 1.55-$\mu$ m InP-based InAs quantum dash lasers, Applied Physics Letters **115**, 091101 (2019).

[32] Y. Matsui, R. Schatz, D. Che, F. Khan, M. Kwakernaak, and T. Sudo, Low-chirp isolator-free 65-GHz-bandwidth directly modulated lasers, Nature Photonics **15**, 59 (2021).

[33] B. Dong, J. Duan, H. Huang, J. C. Norman, K. Nishi, K. Takemasa, M. Sugawara, J. E. Bowers, and F. Grillot, Dynamic performance and reflection sensitivity of quantum dot distributed feedback lasers with large optical mismatch, Photonics Research **9**, 1550 (2021).
